# Analysis of non-retinopathy of prematurity (ROP)–related fundus hemorrhage in preterm infants in China

**DOI:** 10.3389/fped.2022.985268

**Published:** 2022-09-26

**Authors:** Sun Yaoyao, Deng Kaixin, Li Xiaoxin, Zhao Min, Jiang Yanrong, Yin Hong, Qi Huijun, Qian Tong, Linghu Dandan, Yu Wenzhen, Zhao Mingwei, Liang Jianhong

**Affiliations:** ^1^Department of Ophthalmology, Peking University People's Hospital, Beijing, China; ^2^Eye Diseases and Optometry Institute, Beijing, China; ^3^Beijing Key Laboratory of Diagnosis and Therapy of Retinal and Choroid Diseases, Beijing, China; ^4^College of Optometry, Peking University Health Science Center, Beijing, China; ^5^Department of Ophthalmology, Beijing Jishuitan Hospital, Beijing, China

**Keywords:** fundus hemorrhage, new born ocular screening, premature, optometry, retinopathy of prematurity

## Abstract

**Objective:**

To determine the incidence of fundus hemorrhage (FH) not associated with retinopathy of prematurity (ROP) during ocular screening and report their clinical features, risk factors, therapies, and prognosis in a large population of premature newborns.

**Methods:**

The medical records of all preterm newborns admitted to the Department of Ophthalmology, Peking University People's Hospital, Beijing, from January 1, 2016 through August 31, 2021 were retrospectively reviewed. Fundus examinations were carried out by experienced retinal experts. Examination under anesthesia was carried out in newborns with abnormal fundus including vitreous hemorrhage (VH) or retinal hemorrhage (RH) >2 disks' diameter by a Retcam 2 system. A lens-preserving vitrectomy was performed in infants requiring a vitrectomy. A comprehensive medical history was also recorded and analyzed.

**Results:**

During the 5-year period, a total of 7,260 preterm babies were screened. There were 82 (1.13%) newborns and 104 (0.72) eyes with FH, including VH or RH.

Twelve (14.63%) newborns (16 eyes, 15.38%) had VH; 56 (68.29%) (74 eyes, 71.15%) had flame-shaped, superficial hemorrhages; six (7.31%) (6 eyes, 5.77%) had small, round, deep hemorrhages (<2 disk diameters in size); and eight (9.76%) (8 eyes, 7.69%) had large, round hemorrhages (>2 disk diameters). In all, there were 10 (12.20%) cases of intracranial hemorrhage. The mode of delivery was not found to be a significant factor in the occurrence of birth-related retinal hemorrhage (*P* = 0.22).

Six newborns (eyes) with large, round retinal hemorrhage at the posterior pole while the macular was not impacted and 11 cases (15 eyes) with vitreous hemorrhage were required to receive close follow-up with average follow-up time of 105 days. A lens-sparing vitreous surgery was conducted in three patients without any complications.

**Conclusion:**

Preterm newborns with FH that are not caused by ROP are more likely to have superficial, peripheral hemorrhages. Vaginal delivery compression and forceps may be associated with hemorrhage. A lens-preserving vitrectomy is required and considered safe for dense FH involving the refractive media.

## Introduction

Fundus hemorrhage (FH) is an ocular abnormality and one of the important neonatal ocular complications in infants, with an incidence rate ranging from 2.6 to 50.0% (1, 2). There are two main types of neonatal FH—vitreous hemorrhage (VH) and retinal hemorrhage (RH). FH in newborns is caused by a variety of illnesses including retinopathy of premature (ROP), shaken baby syndrome, and systemic coagulation factor abnormalities (2). However, several researchers have confirmed the presence of retinal hemorrhages in healthy babies, which is thought to be birth related (3). In case of significant FH, immediate treatment is sometimes essential to minimize the possible visual development impairment due to FH, such as amblyopia, strabismus, cataract, glaucoma, or other consequences (4, 5). If the FH is not severe, it can be monitored until it absorbs on its own.

In premature newborns, FH in ROP patients have been extensively investigated (6–8). However, FH in premature newborns unrelated to ROP has not been studied as much and includes mostly case series and case reports with no large population-based investigations (4). Thus, little is known regarding the epidemiological characteristics, prevalence of various causes, presenting indications, morphology, distribution, mode of delivery, natural history, prognosis, and possible therapeutic strategies in these newborns.

Considering the possible visual impairment secondary to FH in newborns, early detection and intervention in newborns with FH are critical; however, there are significant challenges involved. Screening all newborns for FH in China is not only difficult to perform but also less efficient, given the current scenario that China is still a developing country (9, 10). Fortunately, extensive fundus screening of preterm infants has been performed in some region. Upon screening, we can discover non-ROP–associated fundus hemorrhage as well as ROP requiring therapeutic intervention in premature newborns during the ROP screening (11, 12). Considering that these lesions are not associated with ROP, or even with preterm birth, analysis of clinical features and risk factors for these FH in premature newborns can be extended to full-term infants. Using these risk factors as a reference and performing fundus screening in full term infants with similar risk factors may prevent poor prognosis because of failure to receive timely treatment.

In this study, we analyzed non-ROP-related FH in all premature newborns during ocular screening at our hospital from 2016 to 2021, and described their clinical features, risk factors, therapies, and prognosis.

## Methods

### Participants

In this retrospective study, the medical records of all preterm newborns admitted to the Department of Ophthalmology, Peking University People's Hospital, Beijing, from January 1, 2016 through August 31, 2021 were reviewed. Consent was obtained from the parents or legal guardians of all newborn patients for inclusion in the study. The study was approved by the ethics committee of Peking University People's Hospital. All newborn patients were referrals from pediatricians or obstetricians in Beijing in accordance to the screening guidelines for ROP in China (13).

Every newborn patient underwent a systemic ocular examination. Outpatient screening was carried out first. The ophthalmologic examinations, which included assessment of the anterior and posterior segments and a red light reflex test were carried out by experienced retinal experts. Fundus examination was made after pupil dilation with an indirect fundoscopy. All the ophthalmologic examinations were recorded carefully. A comprehensive medical history including birth weight, gestational weeks at birth, mode of delivery, history of oxygen inspiration, and the length until follow-up was reviewed. We also reviewed the newborn's health records to see whether there were any complications at birth such as anemia or intracranial hemorrhage. The mother's perinatal complications were also recorded and analyzed.

A specially designed questionnaire recorded all the above mentioned information for all newborns as well as perinatal information for the mothers, and we reviewed the questionnaires for all newborns and collected the results. We performed a detailed retrospective chart review. Based on this, all preterm newborns found to have FH were reviewed. The eligibility criteria were infants whose gestational weeks at birth was <37 weeks and FH not associated with ROP. Newborns with ROP-related fundus changes were excluded. Fundus hemorrhage secondary to additional diseases such as retinal vasculitis and retinoblastoma were also excluded.

### Treatment and follow-up

For newborns with superficial and peripheral RH, further fundus examination was not required; the parents were instead asked to monitor their child's vision development. For other newborns, close ophthalmologic follow-up was required until the RH was totally absorbed. Examination under anesthesia (EUA) was carried out in newborns with abnormal fundus including VH and RH >2 disks' diameter by a Retcam 2 system (Retcam 2, Clarity Medical Systems Inc., Pleasanton, CA, USA). In three patients, we performed a lens-preserving vitrectomy to avoid further complications due to the presence of dense posterior pole FH as soon as the systemic condition was stabilized, as previously described (14). For patients who received vitrectomy, preferential looking and optometry under ciliary paralysis was performed during the follow-up.

### Statistical analysis

Data was analyzed by SPSS software (version 12.0). Statistical comparisons between the newborns with or without fundus hemorrhage were carried out with a chi-square test. The difference between different mode of delivery were carried out with a ANOVA analysis. The statistical significance level was set at *P* < 0.05.

## Results

During the 5-year follow-up period, a total of 7,260 preterm babies were screened. Forty-eight of the tested newborns had VH or RH secondary to active ROP and were thus excluded. Among 82 (1.13%) newborns, 104 (0.72) eyes showed FH (VH or RH), 22 (26.83%) of which were bilateral. Table 1 presents the demographic data for the entire cohort as well as those infants who were diagnosed with FH.

**Table 1:** Demographic data.

	**Total cohort**	**Infants with RH**	**Infants without RH**	**P**
Number of infants	7,260	82	7,178	-
Mean gestational age (weeks)	32.05 ± 1.93	33.14 ± 2.88	31.27 ± 3.26	0.94
No. of males	4,254 (58.59%)	51 (62.19%)	4,203 (58.55%)	0.29
No. of females	3,006 (41.41%)	31 (37.81%)	2,975 (41.45%)	-
Mean age at first examination (weeks)	37.12 ± 4.74	38.90 ± 4.14	36.88 ± 6.03	0.21
Mean birth weight (gm)	1933.23 ± 427.11	1956.79 ± 535.45	1930.34 ± 233.06	0.89
Deed for respiratory support after birth	1,779 (24.51%)	19(23.17%)	1,760 (24.52%)	0.45
Need for resuscitation at birth	1,434 (19.75)	18(21.95%)	1,416 (19.73%)	0.35
Duration of supplemental oxygen (hours)	61.77 ± 0.21	62.33 ± 0.86	59.12 ± 0.15	0.96
Bilateral FH	-	-	22 (21.15%)	-

RH, retinal hemorrhage.

### Morphology and location of fundus hemorrhage

According to the morphological characteristics of FH, 12 (14.63%) newborns (16 eyes, 15.38%) had VH. Vitreous hemorrhage was not dense but dispersed in the vitreous cavity in all newborns but one (1 eye). Among the 70 (85.37%) newborns (88 eyes, 84.62%) with RH, 56 (68.29%) (74 eyes, 71.15%) had flame-shaped, superficial hemorrhages; six (7.31%) (6 eyes, 5.77%) had small, round, deep hemorrhages (<2 disks' diameters in size) at the posterior pole; and eight (9.76%) (8 eyes, 7.69%) had large, round hemorrhages (>2 disks' diameters) located in the posterior pole (Table 2).

**Table 2:** Morphology and location of fundus hemorrhage.

**Location**	**Morphology**	**No. of newborns**	**No. of eyes**	**Total no. of newborns**	**Total no. of eyes**
Vitreous	Dense	1	1	12	16
	Dispersed	11	15		
Retinal	Flame-shaped, superficial hemorrhages	56	74	70	88
	Small, round, deep hemorrhages at the posterior pole	6	6		
	Large, round hemorrhages located in the posterior pole	8	8		
Total No.		82	104		

### Systemic comorbidities in newborns with fundus hemorrhage and their mothers

In all, 47 (57.32%) of neonates with FH had a history of oxygen supplementation, with an average length of 6.03 ± 1.42 days, and 35 (42.68%) neonates had no history of oxygen supplementation. The perinatal history showed that 10 (12.20%) patients had intracranial hemorrhage, 2 (2.44%) had congenital heart disease, 3 (3.65%) had anemia, 3 (3.65%) had hypoxic-ischemic encephalopathy, 3 (3.65%) had hyperbilirubinemia, and 3 (3.65%) experienced intrauterine fetal anoxia. The mode of delivery for all newborns studied is shown in Table 3, together with the related incidence of RH. Despite the fact that the rates of vaginal delivery and forceps use were greater in the group with FH, mode of delivery was not a significant factor in the occurrence of birth-related RH (Table 3, P = 0.22). Pregnancy risk factors were also collected and assessed. Placental abruption (4 cases, 4.9%); placental imprisonment (1 case, 1.22%); diabetes (2 cases, 2.44%); oxygen supplementation during pregnancy (6 cases, 7.3%); and antepartum hormone autacoids administration (3 cases, 3.65%) were among the high-risk pregnancies involving the 82 newborns with FH.

**Table 3:** Mode of delivery of the total newborn cohort.

**Mode of delivery**	**Number of infants (% of total)**	**Number of these without RH (%)**	**Number of these with RH (%)**	***P***
Vaginal delivery	4,503 (62.02%)	4,450 (61.99%)	53 (64.63%)	0.22
Cesarean section	2,508 (34.55%)	2,484 (34.61%)	24 (29.27%)	
Forceps	249 (3.43%)	244 (3.40%)	5 (6.10%)	

RH, retinal hemorrhage.

### Treatment and follow-up

**Figure 1:**
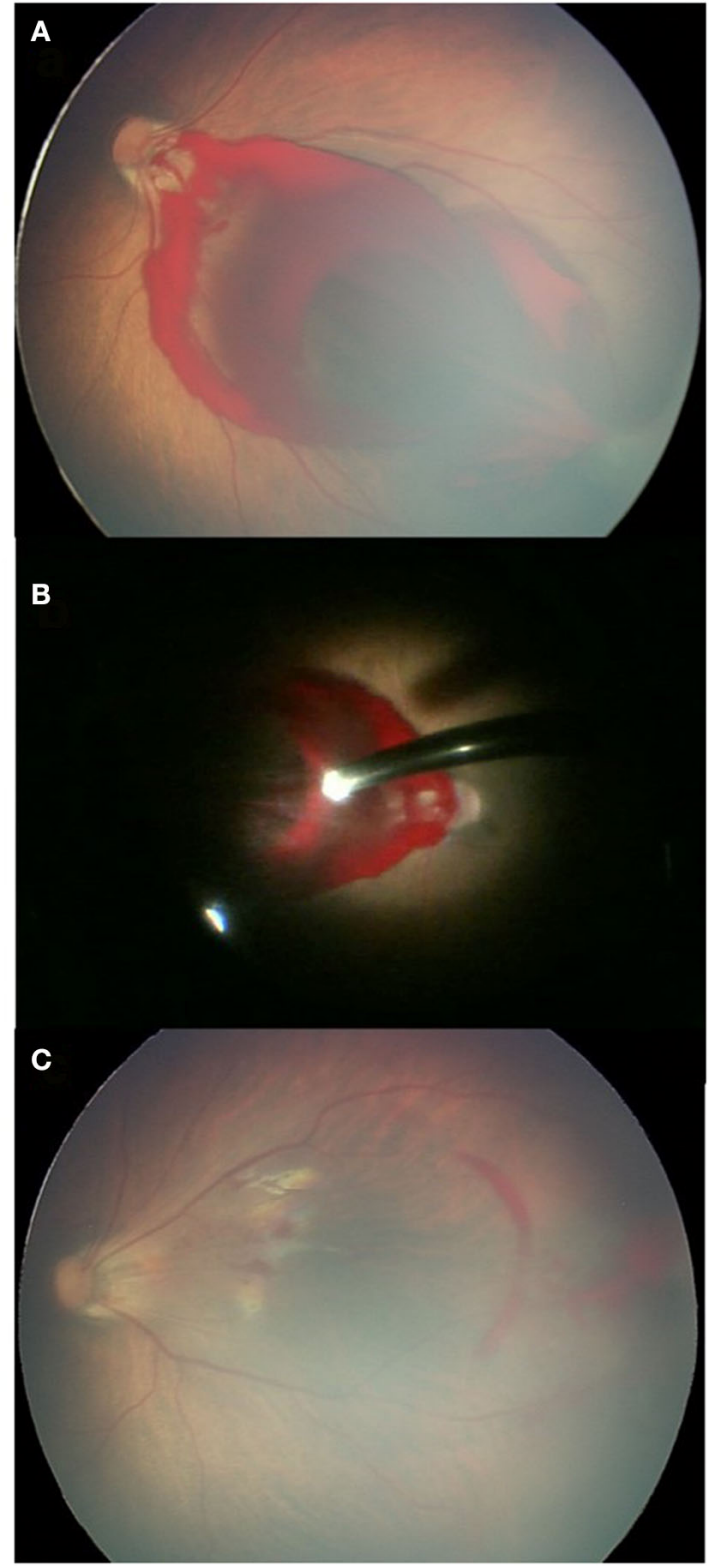
A case of dense RH at the posterior pole. **(A)** Dense RH blocking the macula before surgery; **(B)** what was found during the vitrectomy; and **(C)** the fundus examination 1 month after surgery showing a clear macula.

For different newborns, different management options were selected depending on the morphology and location of FH fundus as stated in the methods. We believed that frequent fundus inspections were not necessary for 56 newborns (74 eyes) with superficial RH, and 6 (6 eyes) with small, round RH; instead, the parents were asked to monitor the child's visual growth. Close follow-up of newborns with less dense FH, including 6 newborns (6 eyes) with large, round RH at the posterior pole (non-impacted macula) and 11 cases (15 eyes) with VH was necessary until the hemorrhage was entirely absorbed. The average time between follow-ups was 105 ± 45.7 days, with intervals ranging from 9 days to 1.5 years. A lens-sparing vitreous surgery was conducted in two cases where the macula was impacted by dense hemorrhage and in one case where the macula was affected by dense vitreous hemorrhage (Figure 1). Patients who had surgery did not develop complications such as retinal detachment, concomitant cataracts, or secondary glaucoma throughout the follow-up period. Details of the newborn who underwent vitrectomy are given in Table 4.

**Table 4:** Clinical data of the three newborns received vitrectomy.

**Case**	**Age at diagnosis (weeks)**	**Sex**	**Preoperative findings**	**Duration between diagnosis and vitrectomy (days)**	**Postoperative SE at last visit**	**Follow-up (months)**	**Preferential looking**
1	35 + 1	F	Dense macular hemorrhage	14	+0.25 DS	18	≥0.053
2	35 + 5	M	Vitreous hemorrhage	10	+1.25 DS	12	≥0.053
3	37 + 5	M	Dense hemorrhage at posterior pole	11	+1.50 DS	15	≥0.053

### Refractive status of newborns who received optometry during the follow up

For newborns who underwent optometry under ciliary paralysis, the results were as follows. At the final follow-up, the mean SE for patients who did not undergo vitrectomy was +1.24 ± 2.06, whereas the mean SE for children who underwent vitrectomy was +1.16 ± 0.66. There was no significant difference between the two groups (*P* = 0.74) (Table 5).

**Table 5:** Refractive status at the last visit of newborns who underwent close follow-up.

	**No. of eyes**	**Average follow-up time (days)**	**Average SE at last visit**	***P***
Without vitrectomy	21	105 ± 45.71	+1.24 ± 2.06	0.74
Vitrectomy	3	450 ± 73.48	+1.16 ± 0.66	

Figure 1 fundus examination of case 1.

## Discussion

In this paper, we reported FH not associated with ROP in a large population. Most cases of FH were superficial RH that did not need further intervention. Although there was no significant association between the mode of delivery and FH, the rates of vaginal delivery and forceps use were greater in the among newborns with FH.

As previously indicated, birth-related RH occur at a rate of 2.6–50%, depending on factors such as inspection time and examination methods. In our investigation, the rate was 1.13%. The intensity of the hemorrhages varied greatly with diverse morphologies of hemorrhage. They are mostly located in the posterior pole and are usually intraretinal, but can also be subretinal or preretinal. Retinal hemorrhages are “typically a mixture of splinter-shaped, flame-shaped, dot and blot hemorrhage, with uncommon sub retinal and preretinal hemorrhage,” per Kaur and Taylor (15). Similarly, Williams et al. (16) discovered flame-shaped hemorrhages (11–20 hemorrhages) as well as isolated and confluent blot hemorrhages. These were frequently >2 disk diameters. However, Forbes et al. reported that birth-related hemorrhages are “minimal in size and number” (17). Our results were consistent with the literature that most newborns had flame-shaped superficial hemorrhages and only few patients had deep, round hemorrhages of varying size.

When we analyzed these risk factors for FH in newborns, we discovered that none of the neonates in our study exhibited systemic coagulation dysfunction, vitamin K insufficiency, or shaken baby syndrome, as previously reported in the literature as possible causes. Based on our findings, a higher percentage of children with FH were born vaginally, particularly those with forceps-assisted delivery, leading us to believe that the etiology of FH in these children was linked to the mode of delivery. Williams et al., for example, discovered a link between the severity of hemorrhage and the mode of delivery, with consecutive vacuum and forceps-assisted deliveries posing the greatest risk (16). Whitby et al. found that “most, but not all” subclinical subdural hemorrhages were associated with instrumental delivery (18). Lindsey et al. found similarities in incidence in relation to mode of delivery to them; however, the number of hemorrhages present were not related to the mode of delivery in their study (19). Although there was no significant association between delivery mode and FH in neonates (*P* > 0.05), vaginal delivery and forceps use was associated with a higher rate of FH than cesarean delivery. Furthermore, we found a higher incidence of intracranial hemorrhage than other complications, which suggested that compression of the eye and brain during delivery is simultaneous. No neonates in this study were delivered with a vacuum based on the following facts: (i) Most obstetricians are aware of the side effects of using vacuum pumps, such as hemorrhage; and (ii) The forceps technique is widely used, technically mature, and with few complications in most hospitals in Beijing.

Close monitoring and observation should be considered for most neonates with FH, because the hemorrhage is usually localized in the peripheral retina, is mostly superficial, and can be quickly absorbed with little effect on the refractive medium. Regardless of the cause of fundus hemorrhage in newborns, there are usually no sequelae. In general, published literature in consistent that birth-related RH clear up quickly (15). Emerson et al., for example, claimed that most hemorrhages resolve on their own within a few weeks (3). Lindsey et al. also discovered that most birth-related bleeding resolved within the first month (19). However, vitrectomy is required if the FH is extensive enough to cause amblyopia or other damage to the retina. Owing to the developed gelatinous nature of the vitreous gel, dense VH in children may take longer to clear spontaneously, resulting in vision loss. Visual deprivation can cause amblyopia and anisometropic amblyopia within days to weeks (20, 21). In a neonate, for example, granular macular alterations have been documented following the clearance of macular hemorrhages (22). Similarly, eyes with a dense VH produced by shaken baby syndrome had a poor visual prognosis in a study by Matthews and Das (23) due to the frequently associated retinal and visual cortex pathologic abnormalities. In our study, optometry was performed on children who underwent vitrectomy. Although there was no significant difference between cases with and without vitrectomy, one patient (case 1) in our study had an SE of only +0.25 DS at 1.5 years of age, suggesting a myopic progression. Regular follow-up is needed to diagnose likely high myopia and amblyopia in the future.

The timing of intervention is mainly determined by the density and duration of the media opacification. For dense FH, vitrectomy is both essential and safe. Antonio Capone et al. (21) documented a group of infants who had amblyogenic VH and/or pre-macular sub-internal limiting membrane hemorrhage due to a variety of factors that might be adequately treated with lens-sparing vitreous surgery. Sayman et al. also established that surgical treatment is more effective at preventing amblyopia and other blood-related disorders. Their average period from diagnosis to surgery was 35–62 days (24). Early vitrectomy may also help to avoid problems like cataracts, epiretinal membrane, retinal detachment, and proliferative vitreoretinopathy that can occur if the VH persists. The duration between diagnosis and vitrectomy was 12 days, 14 days, and 14 days separately in our study. There were no complications such as retinal detachment, secondary cataract, or glaucoma throughout the follow-up period in our study. This result is consistent with previous reports and confirms the safety of vitreous surgery (24).

Our study has some limitations. Screening in this study was only performed in preterm newborns, not full-term infants, owing to the constraints of the screening circumstances. Therefore, it may not be fully representative of the full-term infant population. However, our conclusions can still be extrapolated to all newborns. Furthermore, because this was a retrospective study, the follow-up duration was insufficient. Hence, to track changes in children who have undergone vitrectomy, a longer follow-up period is required in the future.

In conclusion, preterm newborns with FH that are not caused by ROP are more likely to have superficial, peripheral hemorrhages, with birth-related factors being the leading cause. Vaginal delivery compression and forceps use rate were higher in newborns with RH A lens-preserving vitrectomy is required and considered safe for dense FH involving the refractive media. In future, fundus screening may also be required for full-term newborns with similar risk factors.

## Data availability statement

The original contributions presented in the study are included in the article/supplementary material, further inquiries can be directed to the corresponding author.

## Author contributions

All authors listed have made a substantial, direct, and intellectual contribution to the work and approved it for publication.

## Funding

This work was supported by the Program of Development and Cultivation of Medical Innovative Varieties and Industrial Support, Beijing Municipal Science and Technology Commission (Grant Number Z191100007619041), and Beijing Residency Training Quality Improvement Project (No. Zhupei2021043).

## Conflict of interest

The authors declare that the research was conducted in the absence of any commercial or financial relationships that could be construed as a potential conflict of interest.

## Publisher's note

All claims expressed in this article are solely those of the authors and do not necessarily represent those of their affiliated organizations, or those of the publisher, the editors and the reviewers. Any product that may be evaluated in this article, or claim that may be made by its manufacturer, is not guaranteed or endorsed by the publisher.
